# The UK C‐BiLLT: Validity and reliability of an online assessment of spoken language comprehension for children with severe motor disorders

**DOI:** 10.1111/1460-6984.70025

**Published:** 2025-03-18

**Authors:** Lindsay Pennington, Lily Potts, Janice Murray, Johanna Geytenbeek, Kate Laws, Jenefer Sargent, Michael Clarke, John Swettenham, Julie Lachkovic, Catherine Martin, Elaine McColl

**Affiliations:** ^1^ Population Health Sciences Institute, Faculty of Medical Sciences Newcastle University Newcastle upon Tyne UK; ^2^ Faculty of Health and Education Manchester Metropolitan University Manchester UK; ^3^ Department of Rehabilitation Medicine, Amsterdam UMC VU University Amsterdam the Netherlands; ^4^ Regional Communication Aids Service Cumbria, Northumberland, Tyne and Wear NHS Foundation Trust, Newcastle upon Tyne UK; ^5^ Great Ormond Street Hospital NHS Foundation Trust London UK; ^6^ Department of Speech, Language and Hearing Sciences San Francisco State University San Francisco USA; ^7^ Division of Psychology & Language Sciences UCL London UK; ^8^ Communication Aid Service East of England Cambridge University Hospitals NHS Foundation Trust Cambridge UK

**Keywords:** augmentative and alternative communication (AAC), children, measure, reliability, spoken language comprehension, validity

## Abstract

**Background:**

Current UK measures of early spoken language comprehension require manipulation of toys and/or verbal responses and are not accessible to children with severe motor impairments. The Computer‐Based Instrument for Low motor Language Testing (C‐BiLLT) (originally validated in Dutch) is a computerized test of spoken language comprehension that children with motor disorders control using their usual response methods.

**Aims:**

To create a UK version of the C‐BiLLT, evaluate its validity and reliability, and assess its practicability for children with motor disorders.

**Methods & Procedures:**

The C‐BiLLT was translated into British English and items were adapted to ensure familiarity to UK children. A total of 424 children (233 females, 191 males) aged 1:6–7:5 (years:months) without developmental disabilities were recruited from North East England. Children completed the UK C‐BiLLT and Preschool Language Scales 5 (PLS‐5) for convergent validity evaluation and either the visual reception subtest of the Mullen Scales of Early Learning (MSEL) (children aged 1:8–5:5) or Ravens Coloured Progressive Matrices (CPM) (ages 5:6–7:5) to assess divergent validity. A total of 33 children completed the UK C‐BiLLT within 4 weeks of initial assessment for test–retest reliability assessment (intraclass correlation coefficient—ICC). Internal consistency was assessed using Cronbach's alpha and exploratory factor analysis examined structural validity. A total of 24 children (10 female, 14 male; aged 4–12 years) with non‐progressive motor disorders who use augmentative and alternative communication (AAC), rated the UK C‐BiLLT's ease of use and completed British Picture Vocabulary Scales (BPVS) and CPM as for convergent and divergent validity testing.

**Outcomes & Results:**

Internal consistency was high for children without motor disorders (*α* = 0.96). Exploratory factor analysis extracted two factors, together explaining 68% of the total variance. Test–retest reliability was excellent (ICC = 0.95; 0.90–0.98 95% confidence interval—CI). UK C‐BiLLT scores correlated highly with PLS‐5 (*r* = 0.91) and MSEL (*r* = 0.81), and moderately with CPM (*r* = 0.41); and increased across full‐year age‐bands (*F*(6, 407) = 341.76, *p* = < 0.001, *η* ^2^ = 0.83). A total of 19 children with motor disorders rated the UK C‐BiLLT as easy/ok to use; two judged it hard; three declined to rate the ease of use. Their UK C‐BiLLT scores correlated highly with BPVS (*r* = 0.77) and moderately with CPM (*r* = 0.57).

**Conclusions & Implications:**

The UK C‐BiLLT is a valid, reliable measure of early spoken language development and is potentially practicable for children with motor disorders. It may facilitate international research on the language development of children with motor disorders and evaluation of intervention at the national level.

**WHAT THIS PAPER ADDS:**

**What is already known on the subject**

- Young children with motor disorders have difficulties accessing standardized assessments of language comprehension that require children to handle objects or to speak a response.

**What this paper adds to the existing knowledge**

- This study demonstrates the validity and reliability of a UK translation of the C‐BiLLT and suggests that the measure is feasible for children with motor disorders who use AAC and have a reliable method of response via computer access.

**What are the potential or clinical implications of this work?**

- The UK C‐BiLLT is a useful addition to the limited tools currently available to assess early spoken language comprehension of children with motor disorders.

## INTRODUCTION

Cerebral palsy (CP) is the most common cause of non‐progressive motor disorder in childhood, affecting 1.5–2 per 1000 live births (Sellier et al., 2016). Recent research has shown that around 70% of children with CP have communication difficulties (Coleman et al., 2015). One in two children with CP have speech difficulties which limit their intelligibility (often dysarthria) and one in four are non‐verbal (Nordberg et al., 2013). Difficulties with language acquisition affect 60–74% of children with CP (Mei et al., 2016; Vos et al., 2013). The risk of language disorder is hypothesized to be associated with type, distribution and severity of the motor disorder and with non‐verbal cognition (Bax et al., 2006; Choi et al., 2017; Evensen et al., 2023; Mei et al., 2016; Stadskleiv, 2020). One in three children with CP has a cognitive impairment (Himmelmann et al., 2006; Stadskleiv et al., 2018) and language difficulties are more common for these children (Vaillant et al., 2024; Vos et al., 2013). These factors of motor disorder type, distribution and severity and cognition interrelate such that it is difficult to ascertain their interplay in typically small research samples. In turn, assessment for these impairments is challenging. As CP is an umbrella term for non‐progressive motor disorders caused by damage or maldevelopment of the foetal or infant brain (Rosenbaum et al., 2007), the simple risk patterns described above can only loosely inform prognostic advice to parents, with many children presenting with complex characteristics of the traditional typographies thus necessitating specialist assessment.

Norm‐referenced standardized assessments provide a snapshot of children's development with a quantified comparison to their same‐aged peers, and reassessment enables progress to be measured objectively. For children with severe motor disorders, well‐validated norm‐referenced measures of language comprehension help their team to understand children's level of verbal comprehension and any differences between receptive and expressive language. Such information is vital for intervention planning, including the selection and implementation of augmentative and alternative communication (AAC) (Lund et al., 2017; Murray et al., 2019; Webb, Lynch et al., 2019; Webb, Meads et al., 2019). AAC helps people to communicate as effectively as possible, in as many situations as possible and comprises a range of strategies and tools from simple letter or picture boards to sophisticated computer technologies (Communication Matters, 2024). Accurate information on children's language development helps to ensure that AAC systems give children access to the language they need and are neither too linguistically reductive to be interesting or too complex to be useful in everyday life (Judge et al., 2023).

Published norm‐referenced tests of children's language comprehension involve children pointing to pictures or objects, handling objects or speaking a response, all of which may be challenging for children with severe motor disorders. Multiple choice, picture‐based standardized language assessments, such as the Test of Reception of Grammar (TROG) (Bishop, 2003), Peabody Picture Vocabulary Test (PPVT) (Stockman, 2000) and British Picture Vocabulary Scales (BPVS) (Dunn et al., 2009) are completed by children pointing to one of four pictures, either with their finger, hand or eyes. Pictures can be spaced more widely by cutting pages of test booklets, to allow accurate pointing without compromising the standard method of test delivery (Kurmanaviciute & Stadskleiv, 2017). These different methods of direct access have been used in studies involving school‐aged children with motor disorders aged 5 years and above (Hustad et al., 2018; Mei et al., 2016). However, other methods of access such as partner‐assisted scanning in which an adult points to each picture in turn, waiting for a response from the child to indicate their choice adds cognitive processing demands and invalidates standardization (Warschausky et al., 2012).

Whilst multiple‐choice, picture‐based tests of language comprehension for older children, such as the TROG (Bishop, 2003), are often accessible to children with motor disorders, measures for preschool children present challenges as they involve handling objects in response to a command (Geytenbeek et al., 2010). Typically, norm‐referenced standardized language assessments for preschool children involve the use of real objects, often as miniatures, and require children's manipulation of these to respond to commands from the tester, for example, Reynell Developmental Language Scales (RDLS) (Edwards et al., 2011) and The Preschool Language Scale—5th edition (PLS‐5 UK) (Zimmerman et al., 2011). Testing items are designed to mimic play‐based activities that children may be familiar with and are intended to be attractive to look at and touch (DeLuca et al., 2020). In object‐based assessments, children with motor difficulties may be able to indicate their understanding of the language stimulus by changing their method of response. For example, children may be able to eye point to more than one item or to show the examiner where to move an object in response to two or three keyword commands. However, this has limits. For example, ‘put teddy in the box’ cannot be clearly enacted through eye pointing alone since the tester would need to infer the position the child intended (on/in/under). In the UK, speech and language therapists often assess the language comprehension of children with motor disorders informally, through observation, informal testing and reports of performance in unobserved situations (Watson & Pennington, 2015). However, observations of children's performance in daily life lack the controls that standardized tests offer and lack replication over time. Informal assessments of language can be replicated over time and may offer a description of developmental achievements, (for example, comprehension of primary colour names) but do not allow comparison of a child's performance against their peers.

To address the need for a norm‐referenced standardized assessment of early language, Geytenbeek et al. developed the Computer‐Based Instrument for Low motor Language Testing (C‐BiLLT), which children with motor disorders can control using their usual methods of computer access, including direct access using a touch screen or eye pointing and indirect access using switches (Geytenbeek et al., 2014). The C‐BiLLT was developed in Dutch and was based on the language hierarchy of the Dutch version of the New Reynell Developmental Language Scales (NRDLS) (Edwards et al., 2011). The test started with single word noun and verb phrases and progressed through syntactically simple phrases with two and three information‐carrying words to complex phrases referring to two or more concepts and compound sentences, with a total of 75 items. It was validated in the Netherlands with 831 typically developing children aged 1:6–7:6 (Geytenbeek et al., 2014). Examination of the internal consistency showed the items on the C‐BiLLT were cohesive (Cronbach's *α* 0.88) and factor analysis showed that they accessed a single construct that explained 76% of the variance. High correlations of C‐BiLLT scores with scores on other tests of language comprehension (Dutch RDLS *r* = 0.93; PPVT *r* = 0.88) and moderate correlations with scores of tests of the related domain of non‐verbal cognition (Ravens Coloured Progressive Matrices (CPM) *r* = 0.43), indicated the construct validity (convergent and divergent validity) of the C‐BiLLT as a test of spoken language comprehension. Test–retest reliability with the same or different test administrators was excellent (intraclass correlation coefficient (ICC) > 0.95). Differences in C‐BiLLT scores were observed for children between full chronologic years aged 1–7 years and established the norms for Dutch children. Following the initial validation, further studies with children with motor disorders showed that the C‐BiLLT was acceptable and reliably distinguished between children with and without language comprehension difficulties (Geytenbeek et al., 2015). It was noted, however, that a proportion of older children in the samples with and without motor disorders were reaching ceiling on the test. The C‐BiLLT was therefore extended from 75 to 88 items, to add more items to the complex language in the latter part of the test. The 88‐item version of the C‐BiLLT is now used in national epidemiological research and routine clinical practice in the Netherlands (Vaillant et al., 2024).

The C‐BiLLT has been translated into other European languages. Results have been published from the Norwegian version (the C‐BiLLT‐Nor) (Fiske et al., 2020) and the Canadian English version (the C‐BiLLT‐CAN) (Bootsma, Campbell et al., 2023) and are expected soon for studies in Germany, Sweden, Slovenia and Romania. The C‐BiLLT‐Nor and C‐BiLLT‐CAN, have shown similar psychometric properties to Geytenbeek's original version (Bootsma, Campbell et al., 2023; Fiske et al., 2020; Geytenbeek et al., 2014). Scores on both new versions correlated highly with other tests of language comprehension: RDLS (*r* = 0.96 C‐BiLLT‐Nor, *r* = 0.78 C‐BiLLT‐CAN); TROG (*r* = 0.71 C‐BiLLT‐Nor) and PPVT (*r* = 0.84 C‐BiLLT‐CAN), demonstrating good convergent validity. Correlations with the CPM were higher for the C‐BiLLT‐CAN (*r* = 0.87) than for the C‐BiLLT‐NOR (*r* = 0.65) or original C‐BiLLT (*r* = 0.43), possibly a result of the smaller sample size in the C‐BiLLT‐CAN study (C‐BiLLT ‐CAN *n* = 20 versus C‐BiLLT‐Nor; *n* = 122, C‐BiLLT *n* = 120). Both the C‐BiLLT‐Nor and C‐BiLLT‐CAN were found to be reliable, with high test–retest agreement (ICC > 0.8) and standard error of measurement showing that the likelihood that ‘true’ scores fell with ranges of 1.8 to 2.5. Both versions also demonstrated excellent internal consistency (*α* > 0.9), showing a strong relationship between items in the tests. Exploratory factor analysis of the C‐BiLLT‐Nor resulted in a two‐factor solution, rather than the single construct observed by Geytenbeek et al. (2014). Items testing single‐word phrases and simple multiword phrases comprised one factor, and more complex language structures comprised the second, explaining 16.6% and 68.6% of the total variance respectively. The difference between the original C‐BiLLT and the C‐BiLLT‐Nor may have arisen because of the items added to later sections of the C‐BiLLT following its original validation. Like Geytenbeek et al. (2014), Fiske et al. observed a statistically significant difference between full‐year age‐bands on the C‐BiLLT‐Nor, but also on half‐year age bands (*p* < 0.001). The internal structure and comparisons of different age bands are yet to be reported for the C‐BiLLT‐CAN.

This study aimed to create a UK version of the C‐BiLLT, to evaluate its validity and reliability, and to assess its practicability for children with motor disorders. Guided by the Consensus‐based standards for the selection of health Measurement Instruments (COSMIN, 2024), we measured the strength of the relationship between items on the test (internal consistency); the number of different factors assessed by the test and the mapping of items to those factors (internal structure); the relationship between the UK version of the C‐BiLLT and other tests of language (convergent validity) and tests of the related domain of non‐verbal cognition (divergent validity); its ability to differentiate between different levels of language comprehension (discriminant validity); its stability over time (test–test reliability) and the range of scores around which a ‘true’ score may lie (standard error of measurement). Criteria were set for each psychometric property assessed: internal consistency would be high, demonstrated in Cronbach alpha *α* being greater than 0.9; structural validity using factor analysis would show an omnibus measure with all sections of the test included in the factors extracted; construct validity would be demonstrated by correlations of greater than 0.8 between UK C‐BiLLT and a comparator test of language comprehension (convergent validity), moderate correlations (*r* = 0.4–0.7) with a measure of non‐verbal cognition as a related developmental domain (divergent validity), scores would increase with age (discriminant validity); and test retest reliability would exceed 0.75 in ICCs. The psychometric properties of the measure were evaluated with a sample of children who had no known developmental disabilities. Given that the C‐BiLLT, as a computerized, standardized measure, would be a new type of test in the UK which would demand change in assessment practice, we evaluated its practicability through perceptions of the measure's use, time taken to complete the assessment and caregiver ratings of accuracy or results. We also examined the strength of association between scores on the UK C‐BiLLT and on tests of receptive vocabulary and non‐verbal cognition for this group of children, to strengthen our conclusions regarding the psychometric properties of the new measure.

## MATERIALS AND METHODS

### Participants

For the validation of the UK C‐BiLLT we aimed to recruit 480 typically developing children from the North East of England aged 1:6–7:5; 40 per 6‐month age band (from 1:06–1:11 to 7:0–7:5). Children were eligible for inclusion if they had no history of speech or language delay; had no history of visual or hearing impairments (except ear infections), developmental delay or neurological or chronic disorders; and lived with at least one parent and/or caregiver who spoke English at home. The sample size was informed by published language tests (e.g., TROG, Bishop, 2003; and Preschool Language Scales, Zimmerman et al., 1997), which found adequate differentiation between age bands containing 40 children, and by pragmatic considerations. The overall target sample size of 480 children in total, 40 per age band, was deemed adequate for the range of planned analysis (Bujang et al., 2018; Furr, 2021; Hatcher & Stepanski, 1994).

We also aimed to recruit 25 children with motor disorders from North East and Eastern England to test the measure's practicability for use in clinical practice. Inclusion criteria for children with motor disorders were: (a) aged 1:6–12 years (due to their increased risk of delayed language comprehension; Vos et al., 2013); (b) diagnosis of CP or other non‐progressive motor disorder; (c) speech severely limited by motor disorder, with local SLT categorizing their speech as level III (speech not intelligible out of context) or IV (no understandable speech) on the Viking Speech Scale (Pennington et al., 2013); (d) difficulty handling objects, categorized as level III‐V on the Manual Ability Classification System (Eliasson et al., 2006) by their local occupational therapist or physiotherapist); (e) normal hearing; (f) ability to visually inspect and selectively attend to the test materials; (g) at least one parent/caregiver who uses English at home; and (h) an established method of accessing computers/their communication aid (such as using a switch or by eye gaze), so that response method accuracy does not confound validation of the test. Exclusion criteria: speech intelligibility sufficient to answer language assessments using spoken language; no demonstrated response to spoken language in context; cerebral visual impairment.

### Measures

#### Child characteristics

##### Children without motor disorders

Demographic data on each child, comprising their sex (male, female) and age (years:months), were provided by children's school or nursery records, with parental consent. The Income Deprivation Affecting Children Index (IDACI) measures the proportion of children under the age of 16 who live in low‐income households in a local area (English indices of deprivation 2019; opendatacommunities.org). We recorded the IDACI decile for the nursery/school participants who attended.

##### Children with motor disorders

For children with motor disorders, we collected information on children's sex, age, diagnosis and method of accessing their computer and the UK C‐BiLLT from children's medical records. Children's local therapists also rated their speech intelligibility using the Viking Speech Scale, manual function using the MACS, and gross motor function using the Gross Motor Function Classification System (GMFCS) (Palisano et al., 2007). The systems are four‐ or five‐level classification systems, with higher grades indicating greater degrees of impairment.

#### UK C‐BiLLT

The C‐BiLLT was based on the language development hierarchy of the Dutch version of the RDLS. However, although the C‐BiLLT does follow the linguistic hierarchy of the RDLS and includes items that are immediate derivatives of RDLS items, the majority of C‐BiLLT items are new and pertain to objects/situations that are relevant and identifiable to children with severe motor impairment (Geytenbeek et al., 2014). We followed the International Test Commission guidance on translating tests (International Test Commission, 2017). A preliminary translation of the Dutch C‐BiLLT test items into English was completed by an international group including Pennington, Geytenbeek and Bootsma (developer of C‐BiLLT‐CAN version). The UK C‐BiLLT research team further reviewed the UK English C‐BiLLT items to ensure familiarity of expressions and vocabulary to children growing up in the UK, changing images and vocabulary where necessary (e.g., changing chocolate sprinkles to banana). We also cross‐referenced items back to the New RDLS (Edwards et al., 2011), Clinical Examination of Language Fundamental (Semel et al., 2017) and Preschool Language Scales—5th edition (Zimmerman et al., 2011) as robustly validated and widely used assessments of language comprehension to confirm that the linguistic hierarchy of English was retained.

The C‐BiLLT starts with a pre‐test to investigate if a child can reliably communicate a choice between two objects/pictures. The pre‐test has two parts; the first part assesses children's ability to respond to eight familiar objects, and the second part evaluates children's responses to the same eight objects when presented more universally as photographs. The actual computer round consists of 86 items tested via real‐life photographs presented on a computer screen. The draft UK C‐BiLLT retained the pre‐test items (*n* = 2) and 86 computer items translated from the updated Dutch version. As per the original, the 86 items presented on the computer comprised two parts (Part 1: single words; Part 2: phrases) and 12 sections (Table 1). Part 1 has three sections, each comprising 10 items, testing single‐word understanding (nouns [two sections] and verbs [one section]). Items are presented in pairs, with the child selecting their response from a choice of two photographs on the computer screen. Part 2 (56 items in total) comprises nine sections, which increase in phrase length and linguistic complexity. Each item tested has a choice of four photographs. Table 1 shows the overall structure of the computer round, the linguistic structures assessed in each section, the number of items in each section, and example items. We used the same stopping rule of eight consecutive incorrect items as in the original Dutch version of the C‐BiLLT. All children in the study completed the UK C‐BiLLT. All assessors were trained to use the C‐BiLLT by Geytenbeek, its lead developer. Training followed the protocol used to train practising clinicians in the Netherlands and in previous studies of C‐BiLLT translations (Bootsma, Campbell et al., 2023; Bootsma, Stadskleiv et al., 2023; Fiske et al., 2020) and comprised two half‐day sessions on its background, its composition and administration. Assessors were observed administering the test by Geytenbeek prior to its use in the study.

**TABLE 1:** Overview of UK C‐BILLT structure showing language constructs tested in each section, number of items within sections and example items.

Section	Example item	Number of items
*Part 1*		
1. Objects Set 1	Where is the car?	10
2. Verbs	Who is eating?	10
3. Objects Set 2	Where is the dog?	10
*Part 2*		
4. Less frequent objects	Where is the drum?	4
5. Objects, verbs and prepositions	Where do you sleep?	5
6. People performing actions	Who is carrying something?	5
7. Spatial relations and passives	The boy is pushed by the girl	4
8. Prepositions, adjectives	The black key is under the cup	9
9. Active sentences out of context	Who is going to play outside with Anne?	6
10. Two or more concepts	No jars of jam are behind the cloth	9
11. Complex sentences	The banana is on the blue plate and the apple is next to the yellow plate	4
12. Complex‐compound sentences	The food has been served on all but one plate	10

#### Comparator assessments

To assess the UK C‐BiLLT's construct validity we compared the measure with other tests of language comprehension (convergent validity) and non‐verbal cognition, as a related but not directly overlapping developmental domain (divergent validity).

##### Children without motor disorders

For children without motor disorders, the Preschool Language Scales (PLS‐5 UK edition) (Zimmerman et al., 2011) was used as the comparator measure for convergent validity assessment. Two measures of non‐verbal cognition were required for divergent validity assessment to cover the age range of children without motor disorders: The Mullen Scales of Early Learning visual reception scale (MSEL‐VR) (Mullen, 1995) was selected for children aged up to 5:5 years and the CPM (Raven, 1998) for children aged 5:6 years and above.

##### Children with motor disorders

As children with motor disorders have difficulty accessing most standardized tests of language and cognitive development the British Picture Vocabulary Scales (BPVS—3rd edition) (Dunn et al., 2009) and Bayley Scale of Infant of Development Low Motor version (BSID III) (Bayley, 2005) and CPM (Raven, 1998), which can be completed by eye pointing, were selected as the comparator tests (Visser et al., 2014, 2013).

#### Practicability of UK C‐BiLLT for children with motor disorders

To provide an indication of whether the UK C‐BiLLT can be used with children with motor disorders we collected information on the time taken for C‐BiLLT completion (in minutes), ease of use ratings and judgements of the accuracy of results obtained. Children rated how easy the UK C‐BiLLT was to use using a three‐point scale (easy, ok, hard) represented in words and ‘smiley’ face ‘emojis’. Parents/carers/learning support assistants reported if the UK C‐BiLLT result conformed to their view of the child's receptive language development (yes/no; and if no whether the result over or underestimated their child's skill).

### Procedures

The study was approved by the UK Health Research Authority Newcastle and North Tyneside Research Ethics Committee (20/NE/0181).

#### Children without motor disorders

Children without motor disorders were recruited via nine private day nurseries and eight primary schools in North East England. The region has high levels of social deprivation, which is associated with risk to language development (Law J, 2017). In England, children enter school in the academic year in which they turn five. All 3‐ and 4‐year‐olds are entitled to 15 h free education for 38 weeks of the year. To widen access to support and reduce inequalities in children's outcomes, free early education and childcare are also offered to economically disadvantaged 2‐year‐olds, whose families receive state financial aid. We recruited settings serving areas with low, moderate and high levels of deprivation to minimize recruitment bias. Nursery and school managers distributed information sheets about the study to parents of children who were not receiving additional developmental support. Parents/guardians provided written consent for each child to participate.

Assessors who collected data from children without motor disorders were generalist SLTs and SLT students. At each nursery or school site, children were seen individually in a quiet area, where distractions were kept to a minimum. The children completed the PLS‐5 and MSEL VR or CPM prior to the UK C‐BiLLT. The order of PSL‐5 versus MSEL/CPM alternated across children recruited at each site. Children aged up to 3:5 completed Part 1 and Part 2 of the UK C‐BiLLT. Children aged 3:6 and above completed Part 2 only. If any child older than 3:6 failed items in Section 4, Part 1 was administered. Assessments were carried over at two to three sessions, depending on the child's level of attention. Preschool children often completed tests over three sessions, with one assessment per session. Older children sometimes completed the comparator tests in one session with a break to play a game or chat with the examiner in between assessments. The UK C‐BiLLT was completed alone in a final session. Sessions were spread over 2–10 working days, according to the school or nursery's availability. Ten percent of children completed the UK C‐BiLLT a second time 1–4 weeks following their first assessment. Data were collected from February 2022 to March 2023.

#### Children with motor disorders

Children with motor disorders were recruited from two regional communication aid services in England. Parents of children who fit the study criteria were sent letters about the study. Those who were interested contacted the research team to discuss it further and signed written consent forms if they agreed to their child's participation. Children were visited at school or home, at their parent/carer's preference, for data collection visits. Data were collected by highly specialist speech and language therapists who are experienced in assessing the language and communication skills of children with complex communication needs (K.L. and C.M.). Children completed the assessments over multiple sessions, with breaks as necessary. Data were collected from October 2022 to March 2023.

### Analysis

#### Validity and reliability of the UK C‐BiLLT: Analysis of data from children without motor disorders

Descriptive statistics including means and standard deviations of standard scores on PLS‐5, MSEL VR and CPM, and tests of normality (Shapiro–Wilks and Kolmogorov–Smirnov), were used to examine if the data from children without motor disorders were as expected from a typically developing population. The effects of sex and social deprivation (IDACI) on language and non‐verbal cognition were examined using an independent sample *t*‐test and a one‐way analysis of variance (ANOVA), respectively. Each child's response to individual items in the UK C‐BiLLT was recorded as passed or failed. Composite scores were calculated for the full test, Part 1, Part 2 and for each of the 12 sections from the number of correct scores within them.

##### Reliability

Internal consistency, which is the degree to which items on a scale are associated with one another, was measured by Cronbach's alpha (Cronbach, 1951). Cronbach's alpha was calculated for the full test, and for Part 1 and Part 2 separately. Following Nunnally (1970), we took *α* > 0.90 as satisfactory.

Test–retest reliability, referring to the stability of the UK C‐BiLLT scores across administrations completed closely in time, was examined using ICC with two‐way random effects. Levels of agreement were categorized using Koo and Li (2016) whereby ICC 0.5–0.75 = moderate agreement; 0.75–0.9 = good agreement; > 0.9 = excellent agreement.

Standard error of measurement estimates the variation around a ‘true’ score for an individual when repeated measures are taken and are calculated from test–retest ICC and the test's standard deviation.

Reliability and measurement error were calculated for the total UK C‐BiLLT score and each section separately.

##### Validity

Structural validity relates to the dimensionality of an assessment, showing whether it measures single or multiple constructs and from which we can judge fit with hypothesized constructs. Exploratory factor analysis was used to examine the internal structure of the UK C‐BiLLT. Factor analysis assumes that scores on a scale are correlated with each other to some degree but are not co‐linear (*r* = 0.3–0.9) (Hair et al., 2010). As UK C‐BiLLT scores are binary we used point biserial correlations to examine the association between individual items and section composite scores (e.g., item 1 versus score on Section 1; item 1 versus score on Section 2). A total of 84 of 86 computer test items correlated most highly with the section from which they were drawn, confirming that section scores, rather than individual item scores, could be used in factor analysis, which assumes that scores are continuous. The Kaiser–Meyer–Olkin (KMO) measure of sampling adequacy and Bartlett's test of sphericity were calculated to ensure that UK C‐BiLLT section scores were sufficiently correlated (KMO ≥ 0.7; Bartlett Χ *p* < 0.05) to proceed to factor analysis (Hoelzle & Meyer, 2012). Exploratory factor using principal axis factoring with oblique promax rotation, which allows correlations between factors that might be expected in a composite test of language comprehension, was used to examine the dimensionality of the UK C‐BiLLT. Kaiser's criterion of 1 was used to retain components in the analysis, along with visual inspection of Eigenvalue scree plots (Kaiser, 1958).

We measured the relationship between UK C‐BiLLT scores and validated measures of spoken language comprehension and non‐verbal cognition. Convergent validity refers to how closely a measure is associated with other tests of the same construct and was measured through testing the correlation between scores on the UK C‐BiLLT scores (total score including pre‐test and computer test) and on the PLS‐5. Divergent validity refers to the extent to which a measure differs from measures of different constructs and was measured using correlations between UK C‐BiLLT (total score) and tests of non‐verbal cognition (MSEL VR/CPM). We expected higher correlations between UK C‐BiLLT and PLS5 (convergent validity) than between UK C‐BiLLT and MSEL VR/CPM (divergent validity). Scores on the PLS‐5, MSEL VR and CPM were not normally distributed for all age bands, so non‐parametric Spearman rank correlations are reported.

Differences in UK C‐BiLLT scores between age bands (discriminant validity) were estimated using a one‐way ANOVA, with adjustment for IDACI due to the unequal distribution of IDACI categories across age bands. Post hoc tests to compare pairs of age bands were made using Games–Howell tests, which assume variances may be unequal.

A *p*‐value < 0.05 was taken as significant for all tests and results are reported with 95% confidence intervals. Analysis was undertaken using SPSS Version 28 (SPSS, 2021).

#### Practicability of the UK C‐BiLLT Analysis of data from children with motor disorders

Data on children's ratings of the test's ease of use and parents’/carers’/learning support assistants’ ratings of test accuracy and the time taken to complete the test were analysed descriptively. Associations between scores on the UK C‐BiLLT and comparator tests of language comprehension and non‐verbal cognition were calculated for this group of children using Spearman rank correlations.

## RESULTS

### Reliability and validity of the UK C‐BILLT: Results from children without motor disorders

We recruited 444 children without motor disorders for the study. Following discussion with school/nursery staff, 11 children were excluded because of staff concerns about their language/cognitive development. Nine further children were not included: two moved schools; three were ill/absent from school or nursery during data collection periods; two withdrew assent; and two were missing C‐BiLLT data. Of the 424 children included 233 were females, and 191 were males. Data are missing on two children: 1 missing an MSEL result; 1 child missing a CPM result. IDACI scores showed a bimodal distribution, with many children attending schools/nurseries in very low or very high IDACI deciles. We categorized the IDACI scores as low (deciles 1–2), medium (deciles 3–7) and high (deciles 8–10). Table 2 shows the number of children, percentage of males and females, IDACI categorization and mean scores on UK C‐BiLLT, PLS‐5 and MSEL VR/CPM per age band.

**TABLE 2:** Child characteristics and test scores per 6‐month age band.

Age band		Sex		IDACI			UK C‐BiLLT		PLS‐5		MSEL VR/CPM	
(years:months)	*N*	F	M	Low	Med	High	Mean RS	SD	Mean SS	SD	Mean SS	SD
1:06–1:11	31	15	16	58%	13%	29%	27.5	10.7	104.1	16.3	105.8	29.3
2:00–2:05	30	14	16	57%	13%	30%	45.7	7.9	109.2	7.4	107.9^*^	15.9
2:06–2:11	28	15	13	86%	11%	4%	51.2	9.8	102.0^*^	10.8	111.8	28.1
3:00–3:05	40	27	13	40%	30%	30%	54.0	6.9	98.5^*^	11.1	100.2	22.3
3:06–3:11	44	23	21	41%	43%	16%	57.8	5.7	98.9	10.7	102.9	18.7
4:00–4:05	41	25	16	51%	29%	20%	65.0	6.9	98.4	12.3	100.6	18.8
4:06–4:11	34	17	17	56%	27%	18%	65.1^*^	9.5	98.3	13.9	91.0	19.9
5:00–5:05	43	23	20	58%	16%	26%	66.7^*^	5.4	98.0^*^	14.2	93.3^*^	17.4
5:06–5:11	32	14	18	41%	22%	38%	70.7^*^	4.9	103.9^*^	15.3	103.8	11.8
6:00–6:05	43	25	18	63%	16%	21%	72.2	4.6	102.9^*^	12.8	98.1^*^	10.7
6:06–6:11	31	15	16	50%	20%	30%	76.5^*^	5.1	103.6^*^	11.7	99.4	13.0
7:00–7:05	27	20	7	52%	15%	33%	77.6	3.4	103.6^*^	10.8	93.2	14.1
Total	424	233	191	54%	22%	24%			101.4	12.8	MSEL 100.6	MSEL 20.4
											CPM 98.8	CPM 12.6

*Note*: C‐BiLLT, Computer‐Based Instrument for Low motor Language Testing; CPM, Coloured Progressive Matrices; F, female; IDACI, Income Deprivation Affecting Children Index; M, male; MSEL VR, Mullen Scales of Early Learning visual reception scale; PLS‐5, Preschool Language Scales 5; RS, raw score; SD, standard deviation; SS, standard score.

* Scores not normally distributed.

Shapiro–Wilks and Kolmogorov–Smirnov tests indicated that PLS‐5, MSEL VR or CPM raw and standard scores were not normally distributed in seven of the 12 6‐month age bands. Six of the 424 children scored > 1.5 standard deviations (SD) below the mean on the PLS‐5 (standard scores ranging from 69 to 76). A total of 41 children scored > 1.5 SD below the mean on MSEL VR (*n* = 38) or CPM (*n* = 3). Of the 38 scoring > 1.5 SD below the mean on MSEL, 33 were 3–5 years of age, where testing involves picture and letter matching. Low scores may indicate the effect of reduced socialization and education during COVID‐19, and reduced experience of these cognitive tasks compared to the population on which the test was normed. No child scored > 1.5 SD below the mean for their age group on both the PLS‐5 and MSEL VR/CPM.

No effect of sex was observed on standard scores of the PLS‐5 (*t*(422) = 0.50, *p* = 0.62, Cohen's *d* = 0.05), MSEL VR (*t*(291) = 0.13, *p* = 0.62, *d* = 0.18) or CPM (*t*(127) = −0.23, *p* = 0.82, *d* = −0.04). IDACI influenced PLS‐5 standard scores (*F*(2, 420) = 6.89, *p* < 0.001, *η* ^2^ = 0.03) but not MSEL VR (Welch's *t*‐test (2, 136.490) = 1.66, *p* = 0.19, *η* ^2^ = 0.01) or CPM standard scores (*F*(2, 125) = 2.41, *p* = 0.09, *η* ^2^ = 0.04).

Given that the tests were snapshots of the children's performance and no child scored > 1.5 SD below the population mean on both comparator tests, we judged the sample was adequate for examination of the psychometric properties of the UK C‐BiLLT, with adjustment of UK C‐BiLLT scores by IDACI group for analyses by age given impact of IDACI group on the robustly normed PLS‐5.

#### Reliability

Internal consistency was high for the full C‐BiLLT (*α* = 0.96), Part 1 (*α* = 0.94) and Part 2 (*α* = 0.96). Across all three assessments, alpha levels changed by < 0.01 when individual items were deleted.

A total of 52 children were retested on the UK C‐BiLLT, however, only 33 were seen within four weeks of the first test due to school holidays and children's availability. Given that language comprehension may develop rapidly in the preschool years we have confined the reliability analysis to children who were retested less than five weeks after their initial assessment. At least two children in each 6‐month age band were retested. A two‐way random effects model ICC = 0.95 (95% CI = 0.90–0.98), indicated excellent reliability (Koo & Li, 2016) for total UK C‐BiLLT scores. The measurement error for the total score was 2.48. ICCs for individual sections indicated good to excellent agreement, as shown in Table 3.

**TABLE 3:** Test–retest reliability of individual sections on the UK C‐BiLLT.

	Number of items		95% confidence interval		Measurement error
Section		ICC	Lower	Upper	
1. Objects 1	10	0.67	0.32	0.84	0.44
2. Verbs	10	0.99	0.98	0.99	0.15
3. Objects 2	10	0.87	0.74	0.94	0.56
4. Less frequent objects	4	0.98	0.96	0.99	0.12
5. Objects, verbs and prepositions	5	0.83	0.68	0.92	0.53
6. Who questions	5	0.94	0.87	0.97	0.4
7. Spatial relations and passives	4	0.73	0.45	0.87	0.84
8. Prepositions, adjectives	9	0.84	0.67	0.92	1.39
9. Active sentences out of context	6	0.71	0.40	0.86	1.32
10. Two or more concepts	9	0.84	0.68	0.92	1.58
11. Complex sentences	4	0.77	0.53	0.89	0.71
12. Complex‐compound sentences	10	0.70	0.38	0.85	1.36

*Note*: ICC, intraclass correlation coefficient.

##### Validity

**FIGURE 1:**
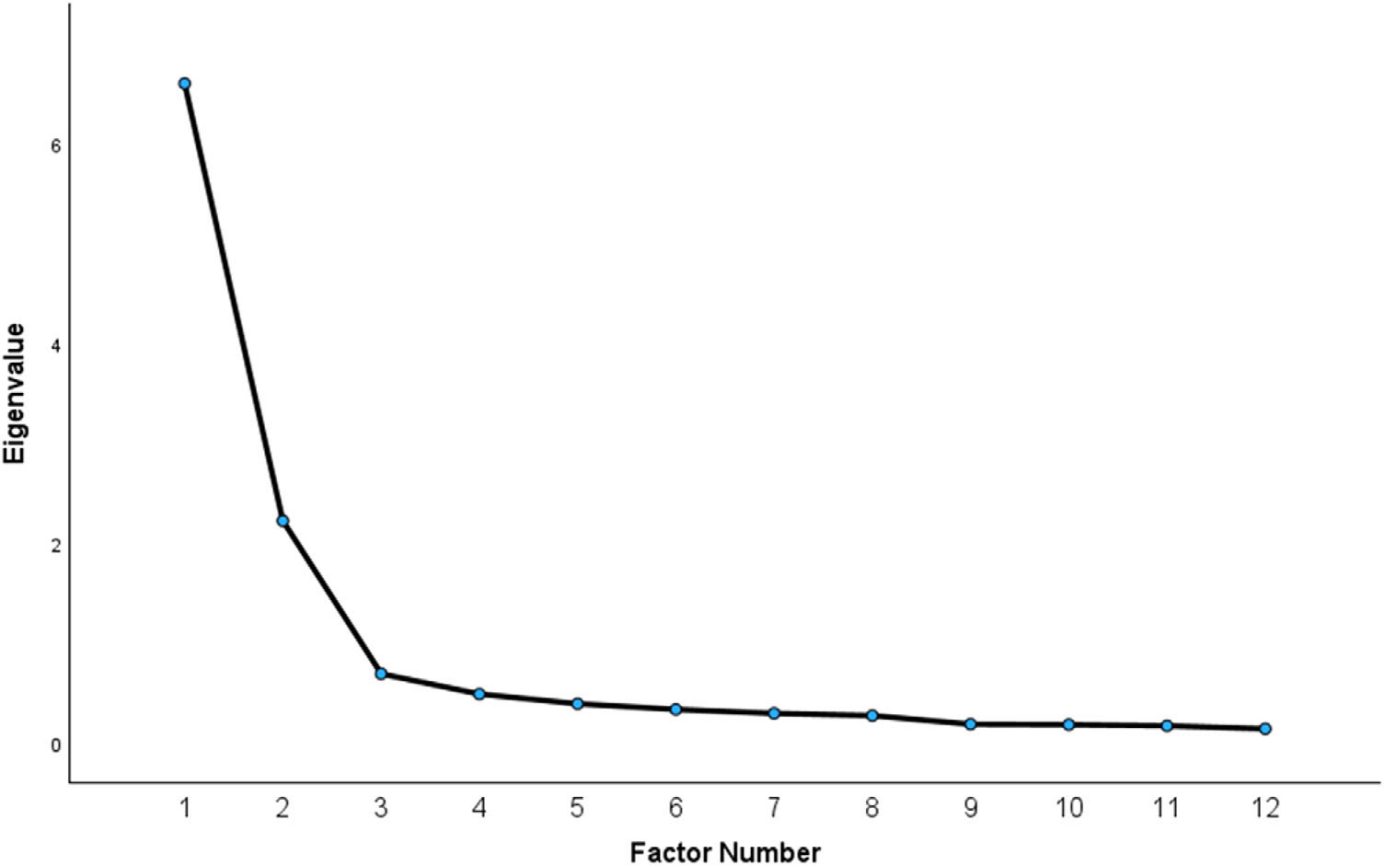
Scree plot of eigenvalues for factors extracted in the exploratory factor analysis.

Preliminary tests showed that assumptions of factor analysis for the examination of the structural validity of the UK C‐BiLLT were met. Correlations of section scores showed that all sections correlated with at least five others, with correlation coefficients between 0.3 and 0.9. The KMO measure indicated sampling adequacy; KMO = 0.92. All KMO values for individual sections were > 0.86; well above the acceptable limit of 0.7 (Kaiser, 1958). Bartlett's test of sphericity (*χ* ^2^ (66) 4155.90, *p* < 0.001) showed that the correlations between sections were sufficient for factor analysis. Principal axis factoring extracted two factors with eigenvalues greater than Kaiser's criterion of 1, which explained 15.86% and 52.46% of the total variance. The scree plot confirmed two clear factors, with factors 3–12 having similar low eigenvalues (Figure 1). Reproduced correlations based on the two‐factor model maintained correlations (*r* = 0.3–0.9) between sections, with each section correlating with at least five others (Table 4).

**TABLE 4:** Reproduced correlation matrix for UK C‐BiLLT sections based on two factor model.

Section	1	2	3	4	5	6	7	8	9	10	11	12
1	**0.44**											
2	0.58	**0.77**										
3	0.54	0.72	**0.68**									
4	0.56	0.74	0.69	**0.72**								
5	0.53	0.71	0.65	0.70	**0.72**							
6	0.53	0.70	0.64	0.70	0.72	**0.72**						
7	0.31	0.41	0.33	0.44	0.54	0.55	**0.63**					
8	0.41	0.55	0.47	0.57	0.67	0.67	0.69	**0.78**				
9	0.24	0.32	0.25	0.37	0.50	0.51	0.67	0.71	**0.73**			
10	0.22	0.29	0.21	0.34	0.49	0.50	0.70	0.74	0.78	**0.84**		
11	0.18	0.23	0.16	0.28	0.42	0.43	0.62	0.65	0.69	0.74	**0.66**	
12	0.16	0.21	0.15	0.25	0.38	0.39	0.55	0.58	0.61	0.66	0.59	**0.52**

Table 5 shows the factor loadings after rotation. Factor 1 includes vocabulary items in Part 1 of UK C‐BiLLT and simple grammatical structures of Part 2. Factor 2 includes more complex grammatical structures and sections requiring complex working memory tasks.

**TABLE 5:** Pattern matrix from the principal axis factoring.

Section	Number of items	Factor 1	Factor 2	Communalities after factor extraction
1. Objects 1	10	0.70		0.44
2. Verbs	10	0.93		0.77
3. Objects 2	10	0.91		0.68
4. Less frequent objects	4	0.85		0.72
5. Objects, verbs and prepositions	5	0.72		0.72
6. People performing actions	5	0.70		0.72
7. Spatial relations and passives	4		0.71	0.63
8. Prepositions, adjectives	9	0.33	0.66	0.78
9. Active sentences out of context	6		0.87	0.73
10. Two or more concepts	9		0.97	0.84
11. Complex sentences	4		0.87	0.66
12. Complex‐compound sentences	10		0.77	0.52
Eigenvalues		1.90	6.30	
% of variance explained		15.86	52.46	

Correlations between the UK C‐BiLLT and PLS‐5 showed a strong association, indicating convergent validity. Correlations between the UK C‐BiLLT and measures of non‐verbal cognition were lower, suggesting divergent validity (Table 6). Correlations between the MSEL and UK C‐BiLLT/PLS‐5 were high. The first half of the MSEL visual reception scale involves handling toys, sorting objects by size and shape and matching pictures. The second half involves discriminating figures, matching letters and fine spatial detail, and is similar to the CPM tasks. To pass these items children need a score of at least 35, which gives an age equivalent of 36 months. In a post hoc investigation, we split the sample into two groups by age (≤ 36 months; ≥ 37 months). Spearman rank correlations showed a stronger association between the MSEL visual reception scale for the younger children than the older children.

**TABLE 6:** Spearman rank correlations between tests of language comprehension and non‐verbal cognition.

Test	*N*	UK C‐BiLLT	PLS‐5	MSEL VR	CPM
*Total sample*					
UK C‐BiLLT	424				
PLS‐5	424	0.91^**^			
MSEL VR	295	0.81^**^	0.87^**^		
CPM	129	0.41^**^	0.27^*^	–	
*≤ 36 months*					
MSEL VR	91	0.75^**^	0.76^**^		–
*≥ 37 months*					
MSEL VR	202	0.58^**^	0.64^**^		–

*Note*: –, No correlation; children were assessed on either MSEL VR or CPM. C‐BiLLT, Computer‐Based Instrument for Low motor Language Testing; CPM, Coloured Progressive Matrices; MSEL VR, Mullen Scales of Early Learning visual reception scale; PLS‐5, Preschool Language Scales 5.

* *p* < 0.01; **p < 0.001.

#### Differences between age bands on UK C‐BiLLT scores

**FIGURE 2:**
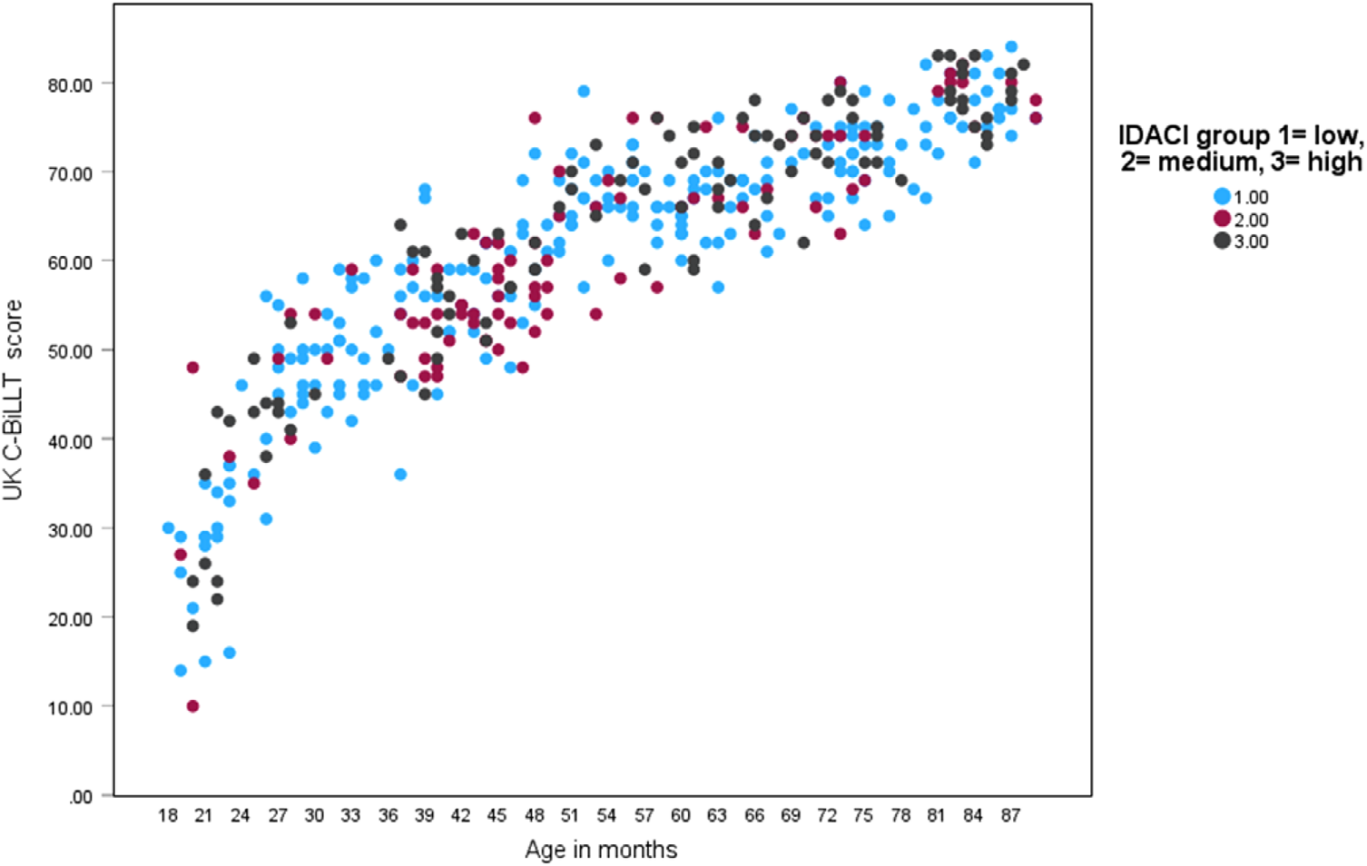
Scatter plot of UK C‐BiLLT score by age (months) by IDACI category. *Note*: C‐BiLLT, Computer‐Based Instrument for Low motor Language Testing; IDACI, Income Deprivation Affecting Children Index.

A scatter plot showed UK C‐BiLLT scores increasing with age, the difference in the distribution of IDACI category across age bands and several outlying scores for children below 6 years of age (Figure 2).

When outliers were removed, a one‐way ANOVA, weighted by IDACI category, revealed that there was a statistically significant difference in UK C‐BiLLT score between 6‐month age bands (*F*(11, 402) = 211.02 *p* = < 0.001, *η* ^2^ = 0.85), but few statistically significant differences between individual pairs of neighbouring age bands. Differences were observed however between full‐year neighbouring age bands (*F*(6, 407) = 341.76, *p* = < 0.001, *η* ^2^ = 0.83), as shown in Table 7. Mean (SD) UK C‐BiLLT scores weighted by IDACI category for full‐year age groups were: 1 year 29.20 (12.31); 2 years = 47.17 (7.65); 3 years = 55.21 (7.62); 4 years = 65.56 (8.12); 5 years = 69.31 (6.82); 6 years = 75.29 (6.46); and 7 years = 77.76 (4.43).

**TABLE 7:** Differences in mean UK C‐BiLLT scores weighted by IDACI category between full‐year age bands.

				95% confidence Interval	
Age comparison	Mean difference	SE	*p*	Lower bound	Upper bound
1:06–1:11 and 2:00–2:11	17.97	1.94	< 0.001	12.10	23.85
2:00–2:11 and 3:00–3:11	8.04	1.05	< 0.001	4.90	11.17
3:00–3:11 and 4:00–4:11	10.35	0.97	< 0.001	7.47	13.23
4:00–4:11 and 5:00–5:11	3.75	0.95	0.002	0.91	6.58
5:00–5:11 and 6:00–6:11	5.98	0.84	< 0.001	3.50	8.47
6:00–6:11 and 7:00–7:05	2.46	0.87	0.074	0.13	5.06

### Practicability of UK C‐BiLLT for children with motor disorders

We recruited 27 children (12 female, 15 male) who had non‐progressive motor disorders and used AAC. Children were aged 4–12 years (mean = 8:02 years). Three children had non‐progressive genetic syndromes; 24 had CP. The speech of 10 children was judged to be unintelligible out of context and was rated III on the Viking Speech Scale (Pennington et al., 2013); 17 had no intelligible speech (Level IV). All children had severe difficulty handling objects (MACS median = IV; range III–V) and used wheelchairs to get around, most had required postural support (GMFCS Median = V; range III–V). Three children refused to undertake the UK C‐BiLLT, BPVS or CPM. Results are presented for the remaining 24 children (10 female, 14 male; mean age 8:03), who completed the UK C‐BiLLT using a range of different access methods.

Five children accessed their computer fitted with a keyguard, four used a touch screen, and one child used a switch; three completed the UK C‐BiLLT via partner‐assisted scanning of the items, and 14 used a combination of eye gaze and partner‐assisted scanning. The mean score for the 24 children with motor disorders on the UK C‐BiLLT was 57 (SD = 19, range = 4–81). They took a mean of 37 min to complete the test (SD = 14; range = 10–65). Their scores on the UK C‐BiLLT correlated strongly with scores on the BPVS (*r* = 0.77, *p* = 0.001) and moderately with the CPM (*r* = 0.57, *p* = 0.01).

A total of 19 children rated the UK C‐BiLLT as easy or ok to use; two judged it to be hard to use; three declined to rate its ease of use. A total of 21 parents/carers/learning support assistants rated the results obtained as an accurate reflection of the child's language comprehension. One was surprised by how many items the child passed but, on reflection, thought the result was accurate. Two parents/carers/learning support assistants judged the result to be an underestimation of the child's comprehension.

## DISCUSSION

This study aimed to assess the validity and reliability of the UK C‐BiLLT on a sample of typically UK‐developing children and its practicability for children with severe motor disorders. Results met the pre‐specified criteria for validity and reliability and an increase in mean scores across full‐year age groups. Children with motor disorders and their carers found the assessment acceptable. The results are discussed in more detail below.

### Reliability

Results from the children without motor disorders suggest that the UK C‐BiLLT has excellent internal consistency and test–retest agreement; and that the assessment is precise with obtained scores varying around a child's ‘true’ score by less than 3 points and less than 3% of the total possible score. Our results are similar to research on other versions of the C‐BiLLT (Bootsma, Stadskleiv et al., 2023; Fiske et al., 2020; Geytenbeek et al., 2014) and suggest that the C‐BiLLT is a reliable measure. The similarity in reliability results across versions of the C‐BiLLT maybe due to the fact that the C‐BiLLT provides observable visual feedback for both the child (test taker) and the test examiner which may improve the reliability of scoring the test items (Geytenbeek et al., 2014).

### Validity

Similar to psychometric testing of other language versions of the C‐BiLLT (Fiske et al., 2020; Geytenbeek et al., 2014), the UK C‐BiLLT scores correlated strongly with a robust measure of language comprehension, the PLS‐5, and moderately with a well‐validated measure of non‐verbal cognition, Ravens CPM. These findings support the construct validity of the UK C‐BiLLT as a measure of language comprehension. As current cognitive/constructive theories consider language as a form of cognition (Chater & Christiansen, 2010; Harris, 2006), some association between language comprehension and non‐verbal cognition is expected. It was noted that the degree of association differed across age groups in our study. The correlation between UK C‐BiLLT and visual reception scale of the MSEL, was high, as too was the correlation between the PLS‐5 and the MSEL. Correlations between MSEL and UK C‐BiLLT/PLS‐5 remained high in post hoc analysis including children aged up to 37 months, who completed object matching and sorting. Correlations were moderate for older children, who were more likely to reach MSEL items that included visual form discrimination, which were similar to the CPM tasks of visual pattern recognition and completion. A similar pattern of higher correlations between C‐BiLLT and other tests of language with non‐verbal cognition for younger children, which decrease with age, was observed by Fiske et al. (2020), for the WPPSI‐IV Block Design subtest (Wechsler, 2013). Fiske et al. propose that such age effects may arise because dimensions of cognitive processing are less differentiated for younger children (Karmiloff‐Smith, 2009; Tideman & Gustafsson, 2004). Our results add support for this hypothesis.

As in other language versions (Bootsma, Stadskleiv et al., 2023; Fiske et al., 2020; Geytenbeek et al., 2014) UK C‐BiLLT scores increased with age, and no floor or ceiling effects were observed, suggesting that the UK C‐BiLLT is suitable for children aged 18 months to 7.5 years of age. ANOVA results showed half and full‐year effects of age on UK C‐BiLLT score, but post hoc analyses showed that only full‐year age band scores were clearly differentiated. The least difference was observed between children of 4 and 5 years of age. Figure 2 shows children aged 48–70 months scoring mid‐60s to around 70 on UK C‐BiLLT. Scores of 65–70 would be achieved by passing most items in sections up to Section 9—active sentences out of context (e.g., Mum is eating Anne's biscuit, who gets upset?) and Section 10—two or more concepts (e.g., A small jar of jam is behind the red jar of jam), assuming that children were not reaching this point in the assessment completely by chance. These sections require the child to make inferences and demand considerable working memory. We did not observe a similar flattening of the growth curve for children aged 48–66 months in our sample on the PLS‐5, on which a linear increase in scores with age across the sample was observed up to around 70 months when many children reached ceiling (see the Supporting Information file online). In the PLS‐5, the total scores of children aged 48–66 months suggested that they were responding correctly to simple sentences containing constructs such as qualifiers (e.g., ‘point to the lorries in order from the smallest to the biggest’) quantity (e.g., ‘point to each animal’), sequence (‘last’) and understanding narrative. The PLS‐5 items may be more like the language heard in everyday life, whereas the UK C‐BILLT items use more formal language. Investigation of the functioning of the individual items in the UK C‐BiLLT is therefore warranted.

Exploratory factor analysis of UK C‐BiLLT scores resulted in two dimensions, with items on Sections 1–6 and 8 loading on one factor and Sections 7–12 loading on a second. Sections loading on the first factor assess single‐word vocabulary, phrases containing one or two keywords, and longer phrases containing prepositions and adjectives. Sections loading on the second factor likewise include longer phrases with prepositions and adjectives but also include spatial relations, inference using knowledge of the world (e.g., Who will play outside with Anne but doesn't yet?), complex grammar with substantial working memory load (e.g., The banana is on the blue plate and the apple is next to the yellow plate). Two factors, with some overlap between sections, were also observed by Fiske et al. in their study of the C‐BiLLT‐Nor (Fiske et al., 2020). Fiske et al. (2020) hypothesized that the overlapping sections that loaded in both factors indicated a unifying dimension of language comprehension. However, the factor loading of the C‐BiLLT‐Nor differed to the current study, with Sections 5 and 6, which assess grammatically simple phrases containing one or two keywords, loading on both factors, rather than Section 8 doing so as in UK C‐BILLT. Section 8 assesses prepositions and adjectives and includes two complex sentences (e.g., ‘only one of the toothbrushes that have been put away has a long handle’ and ‘there are three keys but two have been taken out of the cup’). UK C‐BiLLT results support a hypothesized unifying dimension of language comprehension, but separates vocabulary and simple grammar from complex grammar, rather than vocabulary from grammar as found in the C‐BiLLT‐Nor. Differences between the C‐BiLLT versions may be due to the structure of the two languages being assessed. It will be interesting to see the dimensions of other version of the C‐BiLLT that are currently under development.

Taken together, the factor loading of Section 8 onto both simple and complex grammar understanding and the flattening of scores around 65–70 for children 4 and 5 years of age suggest that further investigation of UK C‐BiLLT item difficulty, particularly for the middle sections, is warranted. We are currently conducting further analysis of the UK children's responses to individual items using item response theory.

### UK C‐BILLT for children with motor disorders

A previous study surveyed 90 clinicians in Belgium, the Netherlands and Norway about their experiences using the C‐BiLLT in practice (Bootsma, Stadskleiv et al., 2023). Despite identifying barriers to its use, such as the need for a stable internet connection and available hardware, the respondents rated the C‐BiLLT highly in terms of appropriateness, acceptability and practicability. Our results support the European findings, suggesting that the UK C‐BiLLT may be acceptable and could be practicable for use in clinical practice, but given the small sample size we cannot yet draw definitive conclusions on the UK C‐BiLLT's feasibility for children with motor disorders. Most parents/carers/learning support assistants thought that the results were an accurate reflection of the child's spoken language comprehension. Most children thought the test was easy to access and completed it in a reasonable amount of time. However, some children required an hour to finish the test. For children with slow movements and/or processing speeds, the UK C‐BiLLT may be better split into two sessions to minimize fatigue and enable accurate completion. The strong association between UK C‐BiLLT and BPVS and moderate association with CPM for children with motor disorders support our conclusion that the measure has good construct validity.

### Strengths and limitations

We tested 424 children, with at least 27 children per 6‐month age band in this validation study. This compares favourably with versions of the test, for example, C‐BiLLT‐Nor and C‐BiLLT‐Can (Bootsma, Stadskleiv et al., 2023; Fiske et al., 2020), who found similar correlations with comparator tests of language and non‐verbal cognition and similar results in age band scores. To our knowledge, this is the first C‐BiLLT study testing the impact of social deprivation on children's language. We used the IDACI decile as a specific measure of household income deprivation relating to childhood. We included children from schools and nurseries across all IDACI deciles, suggesting that results may reflect the breadth of population scores for young children in England from homes with at least one English speaking parent/carer. However, although children in England usually attend schools within their residential neighbourhood, children may cross an area boundary to attend school or nursery their area of residence may have a different level of income deprivation to that of their education setting. Furthermore, individual families within an area will vary in their circumstances and family level of income deprivation may be greater or less than the area mean. Thus, our measure remains a proxy for individual level deprivation and should be interpreted with some caution. A further limitation of our data is that although we sought children with at least one parent who spoke English at home, we did not collect information on the languages spoken; length of exposure to English may have influenced children's responses to the language tests.

Due to the age range of the children included in the study we used two tests of non‐verbal cognition. The CPM was used with only 129 children, which means that the estimates of association with UK C‐BiLLT should be interpreted with caution. Almost 10% of the sample scored greater than 1.5 SD below the mean for their age on the tests of non‐verbal cognition. Low scores may be due in part to children having less access to education and play during COVID‐19. Further validation with populations with more typical developmental and learning experiences may be advised.

Our sample of children with motor disorders was small and restricted to children with an established method of access and who had accurate vision and no hearing impairments. Furthermore, although our inclusion criteria for children with motor disorders comprised aged 18 months to 12 years, all children with motor disorders who were recruited to the study were at least 4 years of age. This could be a product of our recruitment strategy, with children being identified and recruited via tertiary communication aid services. Examination of its use with a larger, representative sample of children who may be referred for AAC, incorporating a wider age range, is now needed to establish the psychometrics of the UK C‐BiLLT for children with motor disorders. Further research should also examine the reliability of the UK C‐BiLLT when used by clinicians without the degree of specialism of the therapists in our study.

## CONCLUSIONS

The UK C‐BiLLT is showing promise as a valid measure of spoken language development. Further investigation of the performance of individual items is now underway to examine if all items should be retained and produce a final version of the measure. The measure fills a gap in the assessment toolbox for the screening of language development of children with motor disorders, particularly in the early stages where accessible tests of vocabulary and simple phrase structures are particularly lacking. For children in the UK who show an understanding of several keywords, the UK C‐BiLLT may be used alongside the TROG, to evaluate comprehension of individual grammatical forms and the complex sentence structures with working memory load. The similarity in results obtained on the UK C‐BiLLT with those of the Dutch, Norwegian and Canadian English versions of the measure shows that we can assess the early language development of children with motor disorders in a similar manner across languages. This will facilitate international research, including epidemiological studies and evaluation of interventions.

## CONFLICT OF INTEREST STATEMENT

The authors declare no conflicts of interest.

## Data Availability

Data collected from children without motor disorders are available at https://doi.org/10.25405/data.ncl.27194901. Data collected from children with motor disorders are not available to prevent identification of participants.
